# Assessing quality of care among maternity waiting home users and non-users in a rural Rwandan hospital

**DOI:** 10.3389/fgwh.2025.1382577

**Published:** 2025-03-18

**Authors:** Edwin Tayebwa, Richard Kalisa, Amedee Fidele Ndibaza, Jeroen van Dillen, Young-Mi Kim, Jelle Stekelenburg

**Affiliations:** ^1^ University Medical Centre Groningen, Department of Health Sciences, Global Health, University of Groningen, Groningen, Netherlands; ^2^ School of Public Health, University of Rwanda, Kigali, Rwanda; ^3^ Department of Monitoring and Evaluation, IntraHealth International, Kigali, Rwanda; ^4^ Amalia Children’s Hospital, Radboudumc Nijmegen, Nijmegen, Netherlands; ^5^ Jhpiego, Johns Hopkins University, Baltimore, MD, United States; ^6^ Department of Obstetrics and Gynecology, Leeuwarden Medical Centre, Leeuwarden, Netherlands

**Keywords:** severe maternal outcomes, maternal near miss, maternal death, adapted WHO maternal near miss criteria, quality of care

## Abstract

Maternal near-miss (MNM) and maternal death (MD) reviews may improve the quality of obstetric care. We assessed the incidence of severe maternal outcomes (SMO) and process indicators among maternity waiting home (MWH) users and non-users in a rural Rwandan hospital. We conducted a retrospective cohort study among women who were eligible for admission to the MWH (users and non-users) at Ruli Hospital in Rwanda and had delivered between January 2015 to December 2019. Using the adapted sub-Saharan Africa (SSA) MNM approach, data for each woman were collected from admission until discharge or death. There were 8,144 deliveries during the study period and 1,305 of them met the criteria for admission at the MWH. There were 326 users and 905 non-users that had live births, respectively. Overall, SMOs were more frequent among MWH non-users [122/905 (13.4%) vs. 8/326 (2.4%) for MWH users]. The leading cause of SMO was post-partum haemorrhage (PPH) (87.5% among MWH users and 45.1% among non-users), followed by sepsis and hypertensive disorders. The MNM incidence ratio was 24.5 for MWH users and 130.4 for non-users. There were four MDs among non-users (MI of 3.3%) due to coincidental conditions and other obstetric complications, and these occurred without admission to the hospital's high dependency unit (HDU). Management of PPH, sepsis and hypertensive complications was optimal. The incidence of SMO was high among MWH non-users. The quality of care in the management of the major causes of SMO was found to be optimal. However, identification and management of coincidental conditions, unanticipated complications of management, and other obstetric complications were not adequate among MWH non-users. There is a need to train health workers to improve the detection and management of these complications to improve quality of care as well as encourage the utilization of MWHs to reduce the burden due to SMO.

## Introduction

Maternal deaths remain high in sub-Saharan Africa (SSA), a region that accounts for roughly 66% of global maternal deaths (1). The major causes of maternal deaths in Rwanda are largely preventable and include postpartum hemorrhage (PPH), hypertensive disorders, abortion, and obstructed labor (2, 3). Rwanda's institutional delivery rate has increased very fast over the past 15 years (i.e., from 28% in 2005 to 93% in 2019/2020) (4) allowing pregnant women to be assisted by skilled birth attendants during labor and delivery. However, this increase may not necessarily translate into improved quality of care at those health facilities since healthcare providers may become overwhelmed due to higher numbers of patients and continue to work in constrained environments, which may increase severe maternal outcomes (mortality and morbidity) (5). In other similar settings, where the implementation of life-saving evidence-based interventions for women coming to deliver at hospitals was not evident, the increased facility delivery rates did not lead to improved quality of care and resulted in severe maternal outcomes (6, 7).

There is evidence suggesting that the review of critical cases, such as maternal near-miss (MNM) and maternal deaths (MD) at the facility level, can improve the quality of facility-based obstetric care (8–11). This approach is widely recognized for monitoring the effective implementation of critical interventions in maternal health and is regarded by the World Health Organization (WHO) as a systematic process for evaluating and improving the quality of obstetric care. To guide the rollout of this process, the WHO, in 2011, developed specific criteria that comprise what is known as the WHO MNM approach. The WHO MNM approach has been used across several countries, especially in low-resource settings, to assess the quality of care. However, a recent systematic review found that the WHO MNM criteria were modified by several researchers to fit low-resource contexts by excluding several laboratory tests and interventions due to resource constraints (12, 13). In 2017, a Delphi study suggested an adapted sub-Saharan (SSA) maternal near-miss tool for low-resource settings that focuses majorly on clinical aspects rather than laboratory criteria (14). Application of the adapted SSA MNM criteria in a resource-limited setting allows for adequate reporting of all cases of severe maternal complications as opposed to using the original WHO MNM criteria that may lead to underreporting as reported in Ethiopia (15). Measurement of severe maternal outcome (SMO) indicators, such as MNM and MD, and process indicators (such as the use of oxytocin to treat PPH) is useful in assessing the quality of obstetric care (16, 17).

To reduce delays in accessing obstetric care, the WHO recommends the establishment and utilization of maternity waiting homes (MWHs) (16). As defined by the WHO, a maternity waiting home is a residential facility, located near a qualified medical facility, where women with high-risk pregnancies can await their delivery and be transferred to a nearby medical facility shortly before delivery, or earlier should complications arise. Maternity waiting homes were recommended as part of a comprehensive package to reduce maternal morbidity and mortality (1). Recent evidence from SSA indicates that utilization of MWHs is associated with improved maternal and perinatal outcomes, including significant reductions in maternal and perinatal mortality (16, 18–21). A recent study on the only MWH in Rwanda showed that mothers who resided in the MWH during pregnancy had better birth outcomes compared to women who did not use the MWH (22). However, the study did not assess the quality of care and so, did not present any evidence of the quality of care provided to mothers delivering at Ruli Hospital (RH). Yet, quality of care is a key factor to consider during the implementation of MWHs (23). Assessing the quality of care among MWH users and non-users is critical as it provides essential information to guide quality improvement in obstetric care and may inform plans to roll out MWHs in Rwanda. Therefore, the objective of the study was to determine the incidence of SMO and process indicators among users and non-users of MWHs in Rwanda.

## Materials and methods

### Study setting

We studied post-partum women who sustained severe maternal complications at Ruli Hospital (RH) in Gakenke District, Rwanda. A detailed description of the study setting has already been published (22). Ruli Hospital lies at 1,788 meters above sea level and is surrounded by a mountainous landscape which characterizes the northern province of Rwanda. With a bed capacity of 179, RH provides comprehensive emergency obstetric and newborn care, an operating theatre, a laboratory, a medical imaging unit, and blood transfusion services for eight health centers and eight health posts. The Hospital is accessible by road although the road network does not reach every community due to steep slopes. Therefore, more than half of the catchment population have to walk for more than one hour to reach a nearby health facility. The maternity staff consisted of one medical officer, five nurses, and nine midwives. The hospital runs the only MWH in the country. The MWH is located within the hospital premises, right near the maternity unit and was constructed in 2011 with support from *Matres Mundi*, an international non-governmental organization.

All services provided at the MWH are covered by health insurance. These services include obstetric care; psycho-social support; and education on nutrition, birth preparedness, as well as breastfeeding. The MWH is currently funded by RH although it still receives individual contributions from some catholic missionaries.

### Study design

This was a retrospective cohort study among women admitted with severe maternal complications and is a continuum of previous studies. Data were collected for the period of January 2015 to December 2019 (22). As published in our previous paper (22), eligibility for admission to the MWH at RH was determined by a medical officer based on the following criteria: a problem related to the pregnancy e.g., history of recurrent abortion; a previous cesarean section scar; previous prolonged labor; problems during the current pregnancy, including preterm premature rupture of membranes, antepartum hemorrhage, reduced foetal movement, pre-eclampsia, etc., and one of the following conditions: at least 36 weeks of amenorrhea, place of residence that was far from the hospital (more than three hours of walking), having no one to take care of the woman at home, a victim of gender-based violence, being in low socio-economic category (“*Ubudehe*”) 1 or 2, as well as clinician's decision.

### Study population

Our study involved two groups of women, MWH users and non-users. Maternity waiting home users were women who resided in the MWH and delivered at RH during the study period, from January 2015 to December 2019. All MWH users met the admission criteria described above. The non-users were women who did not reside in the MWH but delivered at RH during the same period. All non-users could have been users since they met the criteria for admission to the MWH, but they did not reside at the MWH. Using the adapted SSA MNM criteria, data for SMOs were extracted from obstetric records and used to assess the quality of obstetric care provided at RH (14). Women who developed obstetric complications more than 42 days after termination of pregnancy were excluded. The main outcomes were SMO indicators (defined as MNM and MD combined) and process indicators (defined as treatment of PPH, treatment of sepsis, as well as treatment of eclampsia and pre-eclampsia).

### Data collection

Using tablets, relevant data were abstracted from obstetric records by two trained research assistants who were midwives at the same hospital. They visited delivery rooms, obstetric/post-partum wards, maternity waiting rooms, and the gynecology ward to ensure that all client files were brought to the hospital archives before the initiation of data collection. To confirm eligibility, the first two authors, ET and RK, provided supervision and oversight during the selection of obstetric records in the archives and during data abstraction. The abstracted data focused on morbidities, contributory, or associated conditions, as well as treatment or management of complications as seen in Supplementary File S1. From each obstetric file, the following categories of data were collected: socio-demographic characteristics, gestational age, parity, maternal and neonatal outcomes, process indicators for treatment of the main causes of SMO (including use of magnesium sulphate to treat eclampsia, oxytocin to treat PPH, and parenteral antibiotics to treat sepsis.

### Data processing and analysis

The data were cleaned using MS Excel 2016 and analysed using STATA 15. Descriptive statistics using frequencies and percentages were utilized to calculate outcome indicators. Results were presented in tables, in accordance with the adapted SSA MNM tool. Indicators, such as MNM ratio, mortality index, SMO ratio, hospital access, and process indicators were calculated for both MWH users and non-users. Definitions of outcome indicators are presented in Supplementary File S2.

### Ethical considerations

The Rwanda National Ethics Committee approved the study (protocol code 335/RNEC/2020).

## Results

Socio-demographic characteristics of MWH-users and non-users were already published (22). The mean age for MWH users was 29 years while that for non-users was 30. Most women were subsistence farmers and had health insurance. A total of 8,144 women gave birth during the study period. Of these, 1,305 met the admission criteria for admission at the MWH, including 329 MWH users and 976 non-users. Of these, 326 users and 905 non-users had live births, respectively. Overall, SMO occurred in 8/326 (2.5%) of MWH users (8 MNM and 0 MD) and 122/905 (13.5%) of (118 MNM and 4 MD) non-users (Table 1). The observed difference in the occurrence of SMO in the two groups was significant as not using the MWH was associated with more SMO [Chi^2^ (1): 30.9, *p* < 0.001].

**Table 1:** Morbidities among women with severe maternal outcomes.

Morbidity	MWH users 8 (%)	MWH non-users 122 (%)
1. Women with severe complications		
Severe postpartum hemorrhage	7 (87.5)	55 (45.1)
Severe pre-eclampsia	0 (0.0)	3 (2.5)
Eclampsia	0 (0.0)	4 (3.3)
Sepsis or severe systemic infection	1 (12.5)	12 (9.8)
Ruptured uterus	0 (0.0)	9 (7.4)
Other complications associated with the management of SMO	0 (0.0)	39 (32.0)
2. Organ dysfunction in maternal near-miss cases		
Cardiovascular dysfunction	7 (87.5)	116 (98.3)
Respiratory dysfunction	1 (12.5)	0 (0.0)
Others	0 (0.0)	2 (1.7)
3. Organ dysfunction in maternal deaths		
Cardiovascular dysfunction	0 (0.0)	2 (50.0)
Respiratory dysfunction	0 (0.0)	2 (50.0)
Renal dysfunction	0 (0.0)	0 (0.0)

### Morbidities among women with severe maternal outcomes

Morbidities among MWH users and non-users are presented in Table 1. Among women with severe complications, severe PPH occurred frequently (i.e., in 87.5% of MWH users and 45.1% of non-users). This was followed by sepsis (12.5% among MWH users and 9.8% among non-users). There were no cases of pre-eclampsia and eclampsia among MWH users while among non-users, it was 2.5% and 3.3%, respectively. The most common organ dysfunction in MNM was cardiovascular dysfunction which affected 87.5% of MWH users and 98.3% of non-users. For organ dysfunction in MD, non-users had cardiovascular (50%) and respiratory (50%) dysfunction while there were no MD among MWH users.

### Causes of severe maternal outcomes

Post-partum hemorrhage was the leading cause of MNM among MWH users (87.5%), corresponding to a MNM ratio per 1,000 live births of 21.5. This was followed by unanticipated complications of management (12.5%) with a MNM ratio per 1,000 live births of 3.1. The leading cause of MNM among non-users was unanticipated complications of management (40.0%), with a MNM ratio of 43.1. This was followed by PPH (37.5%) with a MNM ratio of 50.8; obstetric complications (21.3%) with a MNM ratio of 28.7; hypertensive disorders (6.6%) with a MNM ratio of 8.8; and coincidental conditions (3.3%) with a MNM ratio of 4.4.

Maternal deaths occurred among non-users only. Coincidental conditions (75%) and other obstetric complications (25%) were the causes of MD. The mortality index (MI) due to obstetric complications was 3.7 while that of coincidental conditions was 42.9. Having had a previous cesarean section was the leading contributor to MNM among MWH users (4/8; 50%), followed by prolonged labor (2/8; 25%) and anemia (2/8; 25%). Anemia was the leading factor associated with MNM among non-users (46.7%) followed by previous cesarean sections (27%), prolonged labor (18%), coagulation disorders (7.4%) and HIV infection (0.8%) (Table 2).

**Table 2:** Underlying causes of severe maternal outcomes.

Causes of SMO	Maternal near miss		Maternal death		MNMR^a^/1,000 live births		MI^b^ (%)	
	MWH users (*N* = 8)	MWH non-users (*N* = 122)	MWH users (*N* = 0)	MWH non-users (*N* = 4)	MWH users	Non-users	MWH users	Non-users
	*n*/*N* (%)	*n*/*N* (%)	*n*/*N* (%)	*n*/*N* (%)				
Underlying causes								
Post-partum hemorrhage	7 (87.5)	46 (37.5)	0 (0.0)	0 (0.0)	21.5	50.8	0.0	0.0
Other obstetric disease/complications	0 (0.0)	26 (21.3)	0 (0.0)	1 (25.0)	0.0	28.7	0.0	3.7
Unanticipated complications of management^c^	1 (12.5)	39 (31.9)	0 (0.0)	0 (0.0)	3.1	43.1	0.0	0.0
Hypertensive disorders	0 (0.0)	8 (6.6)	0 (0.0)	0 (0.0)	0.0	8.8	0.0	0.0
Coincidental conditions	0 (0.0)	4 (3.3)	0 (0.0)	3 (75.0)	0.0	4.4	0.0	42.9
Pregnancy-related infections	0 (0.0)	0 (0.0)	0 (0.0)	0 (0.0)	0.0	0.0	0.0	0.0
Medical/surgical/mental disease or complication	0 (0.0)	0 (0.0)	0 (0.0)	0 (0.0)				
Unknown	0 (0.0)	0 (0.0)	0 (0.0)	0 (0.0)				
Contributory causes/associated conditions								
Anemia	2 (25.0)	57 (46.7)	0 (0.0)	0 (0.0)	6.1	63.0	0.0	0.0
Previous cesarean section	4 (50.0)	33 (27.0)	0 (0.0)	0 (0.0)	12.3	36.5	0.0	0.0
Prolonged labor	2 (25.0)	22 (18.0)	0 (0.0)	2 (50.0)	6.1	24.3	0.0	8.3
Coagulation disorders	0 (0.0)	9 (7.4)	0 (0.0)	1 (25.0)	0.0	9.9	0.0	10.0
HIV infection	0 (0.0)	1 (0.8)	0 (0.0)	1 (25.0)	0.0	1.1	0.0	50.0

^a^ Maternal near miss ratio.

^b^ Mortality index.

^c^ Such as complications resulting from blood transfusion and allergy to antibiotics.

### Severe maternal outcomes, overall near-miss indicators, and facility-related indicators

As seen in Table 3, among MWH users, there were 8 cases of MNM translating to an MNM ratio of 24.5 per 1,000 live births. From the MWH non-users, there were 118 cases of MNM and the MNM ratio was 130.4 per 1,000 live births. There were 4 MDs among non-users, translating to an MI of 3.3%.

**Table 3:** Severe maternal outcomes, near-miss and facility-related indicators.

Indicators	MWH users	MWH non-users
All deliveries	**329**	**976**
All live births in the population	326	905
Severe maternal outcome (SMO) cases (n)	8	122
Maternal deaths	0	4
Maternal near miss	8	118
Overall MNM indicators		
SMO ratio (per 1,000 live births)	24.5	134.0
MNM incidence ratio (per 1,000 live births)	24.5	130.4
MNM mortality ratio (per 1,000 live births)	24	131
Mortality index (%)	0	3.3
Hospital access indicators		
High dependence unit use		
HDU admission rate (%)	0.6 (2/329)	0.9 (9/976)
HDU admission rate among women with SMO (%)	25.0	7.6
MD assisted without HDU admission (%)	0.0	100.0

The admission rate in the high dependency unit (HDU) among all MWH users and all non-users was 0.6 and 0.9, respectively. The HDU admission rate among women with SMO was 25% for MWH users and 7.6% for non-users. All four deaths occurred without admission to HDU (Table 3).

### Process indicators

As seen in Table 4, to manage severe maternal complications, 100% of MWH users and 96.4% of non-users, respectively, received oxytocin to prevent and/or treat PPH. In addition to receiving oxytocin, 57.1% (4/7) of MWH users and 78.2% (43/55) of non-users received blood transfusions. Other forms of management of PPH included laparotomy (15.6% for non-users), hysterectomy (3.6% for non-users), and admission in HDU (42.8% for MWH users and 3.6% for non-users). Maternity waiting home users did not undergo hysterectomy or laparotomy as part of the management of PPH. Also, our results indicate that there was no case of eclampsia or severe pre-eclampsia among MWH users and so none of them received anticonvulsants. However, among non-users, 7 women had eclampsia and severe pre-eclampsia and 100% of them received magnesium sulphate. To manage puerperal sepsis, 91.6% of MWH non-users with sepsis received parenteral antibiotics. One MWH user developed sepsis, and this was managed with laparotomy.

**Table 4:** Process indicators related with specific conditions.

Indicator	MWH users (8)	MWH non-users (122)
	*n* (%)	*n* (%)
Treatment of severe postpartum hemorrhage		
Target population: women with severe PPH	7	55
Oxytocin use (treatment of PPH)	7 (100.0)	53 (96.4)
Other interventions (e.g., admission to HDU)	3 (42.8)	2 (3.6)
Blood transfusion	4 (57.1)	43 (78.2)
Laparotomy	0 (0.0)	8 (15.6)
Hysterectomy	0 (0.0)	2 (3.6)
Use of anticonvulsants for eclampsia		
Target population: women with eclampsia	0	7
Magnesium sulphate	0 (0.0)	7 (100.0)
Other anticonvulsants	0 (0.0)	0 (0.0)
Treatment for sepsis		
Target population: women with sepsis	1	12
Parenteral therapeutic antibiotics	0	11 (91.6)
Laparotomy	1 (100.0)	2 (16.7)

## Discussion

We studied the quality of maternity care at Ruli Hospital in Gakenke District, Rwanda, by assessing the incidence of SMO and process indicators among users and non-users of maternity waiting homes. Overall, SMO occurred in 8/326 (2.5%) of MWH users and 122/905 (13.5%) of non-users. Our findings indicate that the high prevalence of MNM amongst MWH non-users lies within the wide range of ratios reported in studies from other low-income countries (13, 14, 24). The high MNMR of 130.4 per 1,000 live births among MWH non-users (compared to 24.5 per 1,000 live births among MHW users) may be explained by the delayed arrival of the women at the hospital because they had to travel long distances – since they did not reside in the MWH due to the perception that the cost of their stay at the MWH would be high. Other reasons why women did not stay at the MWH included lack of family support and limited awareness about the existence of the MWH (25). It was not surprising that MWH users in our study had a lower MNMR compared to MWH non-users because they (MWH users) were closely monitored and received immediate care while in the MWH to prevent complications. The MI among MWH non-users (3.3%) was comparable to that reported by other studies in middle-income countries (13, 26, 27). The high MNMR, coupled with MI comparable to that of middle-income countries, could be a demonstration that, despite the high caseload, the hospital is able to provide adequate care and timely referrals as needed. It is worth noting that RH provides pre-referral care to ensure that their critical patients are stabilized before referring them to Kigali University Teaching Hospital (CHUK) for specialized care. The fact that no MD was reported among MWH users (MI of 0.0%) could indicate early detection and management of complications for women residing in the MWH. Also, as published in other studies, there is improved availability of blood, medicines, on-site mentorship by professional association mentors, health insurance and social behavioral change messages all of which contribute to the prevention and better management of obstetric complications (24, 26), and this may further improve outcomes for MWH users.

The main direct causes of SMO were severe PPH, sepsis, uterine rupture, and hypertensive disorders, and this was comparable to other studies in low-resource countries (24, 28, 29), although the latter did not focus on MWH users and non-users. Nonetheless, our findings show that all these morbidities occurred more frequently among MWH non-users than users, indicating a potential benefit of staying in MWHs before delivery (16, 22). Particular attention is needed for conditions with a high MI, in particular coincidental conditions and other obstetric complications that accounted for the four MDs among MWH non-users. These complications were previously reported in studies at a tertiary hospital in Rwanda (29, 30). The relative inexperience of medical officers working in district hospitals may compound this problem (30), combined with improper use of anesthetic drugs as well as inadequate skills in advanced life support in obstetrics (30), hence a need for tailored training to close such knowledge and skills gaps. Training is very critical because our study also found a high number (higher than what was found in other studies) (7, 31), of unanticipated complications of management among MWH non-users (31.9%) which further underscores the need to improve the identification and management of obstetric emergencies monitoring of patients receiving critical care, such as blood transfusion, to avoid complications of management. This reinforces the need to train health providers on safe blood practices and ensure use of national blood transfusion protocols in obstetrics (32, 33). Moreover, we also noted that current guidelines on MNM do not provide a standard definition for unanticipated complications of management, yet this would be important especially in contexts where the quality of obstetric care is still inadequate. It would therefore be of benefit to standardize the definition of unanticipated complications of management to enable resource-limited contexts to investigate and identify such complications to improve obstetric outcomes.

Ruli Hospital does not have an intensive care unit (ICU). So, critically ill patients are either admitted to HDU for close monitoring or transferred to a tertiary hospital for specialized care. We reported that the HDU admission rate for women with SMO was 25% for MWH users and 7.6% for non-users. It was surprising to note that MWH non-users had a lower HDU admission rate, yet, they had a higher burden of SMO. Considering the WHO range (3%-5%), HDU use at RH is high and indicates overuse of the facility by MWH users. This could be because the MWH is located within the hospital, close to the HDU, which improves access for MWH users. All four MDs that occurred among non-users were not admitted to HDU and this is consistent with a study in Zimbabwe where most (88.8%) of MDs occurred without ICU admission (27). It is therefore critical to improve admission to HDU for MWH non-users experiencing serious complications, considering the high burden of SMO, including conditions such as organ dysfunction that requires close monitoring.

It is important to note that there were no cases of hypertensive disorders (pre-eclampsia or eclampsia) among MWH users. Health workers visit MWH users twice daily to monitor their vital signs, including blood pressure, and any abnormal findings are reported, investigated, and managed accordingly, which may explain why MHW users did not develop hypertensive complications. Among MWH non-users, our findings revealed optimal care for all (100%) women with hypertensive complications. This is comparable with findings from a similar study in another Rwandan hospital although the latter did not focus on MWHs (28). This reflects timely diagnosis and treatment of pre-eclampsia and eclampsia, even for women who do not reside at the MWH before delivery. In Rwanda, 98% of pregnant women are seen by a skilled provider during antenatal care (4), and screening for hypertensive disorders during pregnancy is systematically done during antenatal care visits. Therefore, MWH non-users may have already been screened and diagnosed by the time they came to the hospital. In our study, the management of PPH and sepsis at RH was higher compared to what was reported in another study (30). This may reflect adherence to treatment protocols by health workers at RH.

This is the first study in Rwanda to utilize the adapted WHO MNM criteria for SSA to assess the quality of care among MWH users and non-users. The study provides valuable insights into the quality of maternity care at RH, with a focus on MWHs. While the study has contributed significantly to our understanding of the quality of care in the context of MWHs, it is essential to acknowledge its limitations, including a small sample size for MWH users with SMO which made it impossible to compare findings between users and non-users. In addition, we relied on retrospective data, and this is prone to underestimation due to limited post-discharge follow-up of mothers (up to 42 days) by RH. To minimize the effect of these limitations, we spread out the study period to cover five years so that we capture as many eligible mothers as possible. Results from this study may only be generalized in similar rural contexts in Rwanda. Future research directions could include integrating the use of the international classification of disease codes (11th version) into the WHO MNM criteria to improve standardization, developing an integrated module for resource-limited health facilities and assessing the specificity, sensitivity, and predictive value of these adapted tools (13).

## Conclusion

We observed a higher incidence of severe maternal outcomes among maternity waiting home non-users than maternity waiting home users (13.5% vs. 2.5%). The major causes of severe maternal outcomes were post-partum hemorrage, unanticipated complications of management, obstetric complications, and hypertensive disorders. Despite the optimal care provided to manage the major causes of severe maternal outcomes, findings also revealed suboptimal care in the identification and management of coincidental conditions and other obstetric complications among maternity waiting home non-users which resulted in maternal deaths. Our findings underscore the need to train health workers to improve detection and management of obstetric complications. Due to the observed potential benefit of reducing the burden due to severe maternal outcomes, utilization of maternity waiting homes should be encouraged to improve maternal outcomes.

## Data Availability

The raw data supporting the conclusions of this article will be made available by the authors, without undue reservation.
