# Supplementary material for: Assessing quality of care among maternity waiting home users and non-users in a rural Rwandan hospital
Source: Front Glob Womens Health. 2025 Mar 18;6:1382577. doi: 10.3389/fgwh.2025.1382577 (PMC11959047; doi:10.3389/fgwh.2025.1382577)
Supplement: Supplementary file 1 [file Table1.docx]

**Supplementary file S1: Data collection tool**

<https://ee.kobotoolbox.org/x/zm5HWM6y>
