# Supplementary material for: Assessing quality of care among maternity waiting home users and non-users in a rural Rwandan hospital
Source: Front Glob Womens Health. 2025 Mar 18;6:1382577. doi: 10.3389/fgwh.2025.1382577 (PMC11959047; doi:10.3389/fgwh.2025.1382577)
Supplement: Supplementary file 2 [file Table2.docx]

**Supplementary file S2: Definition of maternal near-miss and process indicators**

**Maternal near-miss indicators**

- **Maternal near-miss** (MNM) referred to a woman who nearly died but survived a complication that occurred during pregnancy, childbirth or within 42 days of termination of pregnancy.
- **Maternal death** (MD) was the death of a woman while pregnant or within 42 days of termination of pregnancy or its management, but not from accidental or incidental causes.
- **Live birth** (LB) referred to the birth of an offspring which breathes or shows evidence of life.
- **Severe maternal outcome** (SMO) referred to all maternal deaths and maternal near-miss cases.
- **Severe maternal outcome ratio** (SMOR) referred to the number of women with SMO (MNM + MD) per 1000 live births (LB).
- **MNM ratio** (MNMR) referred to the number of MNM cases per 1000 live births (MNMR = MNM/LB).
- **Maternal near-miss mortality ratio** (MNM : 1 MD) referred to the ratio between MNM cases and maternal deaths.
- **Mortality index** referred to the number of maternal deaths divided by the SMO expressed as a percentage [MI = MD/(MNM + MD)].

**Process indicators**

- **Treatment of severe postpartum hemorrhage** was calculated as the number of women with postpartum hemorrhage who received therapeutic oxytocin divided by the number of all women with postpartum hemorrhage.
- **Management of eclampsia/pre-eclampsia** was calculated as the number of women with eclampsia/pre-eclampsia who received magnesium sulfate divided by the number of all women with eclampsia.
- Treatment of sepsis was calculated as the number of women with severe systemic infections or sepsis who received antibiotics divided by the number of all women with severe systemic infections or sepsis.
